# Integrated Genomics Identifies Convergence of Ankylosing Spondylitis with Global Immune Mediated Disease Pathways

**DOI:** 10.1038/srep10314

**Published:** 2015-05-18

**Authors:** Mohammed Uddin, Dianne Codner, S M Mahmud Hasan, Stephen W Scherer, Darren D O’Rielly, Proton Rahman

**Affiliations:** 1 Genetics and Genome Biology, The Hospital for Sick Children, Toronto, Ontario, Canada; 2 Faculty of Medicine, Memorial University, Newfoundland, St. John’s, Canada; 3 McLaughlin Centre, University of Toronto, Toronto, Ontario, Canada; 4 Department of Molecular Genetics, University of Toronto, Ontario, Canada

## Abstract

Ankylosing spondylitis(AS), a highly heritable complex inflammatory arthritis. Although, a handful of non-HLA risk loci have been identified, capturing the unexplained genetic contribution to AS pathogenesis remains a challenge attributed to additive, pleiotropic and epistatic-interactions at the molecular level. Here, we developed multiple integrated genomic approaches to quantify molecular convergence of non-HLA loci with global immune mediated diseases. We show that non-HLA genes are significantly sensitive to deleterious mutation accumulation in the general population compared with tolerant genes. Human developmental proteomics (prenatal to adult) analysis revealed that proteins encoded by non-HLA AS risk loci are 2-fold more expressed in adult hematopoietic cells.Enrichment analysis revealed AS risk genes overlap with a significant number of immune related pathways (*p* *<* 0.0001 to 9.8 × 10^-12^). Protein-protein interaction analysis revealed non-shared AS risk genes are highly clustered seeds that significantly converge (empirical; *p* *<* 0.01 to 1.6 × 10^-4^) into networks of global immune mediated disease risk loci. We have also provided initial evidence for the involvement of *STAT2*/*3* in AS pathogenesis. Collectively, these findings highlight molecular insight on non-HLA AS risk loci that are not exclusively connected with overlapping immune mediated diseases; rather a component of common pathophysiological pathways with other immune mediated diseases. This information will be pivotal to fully explain AS pathogenesis and identify new therapeutic targets.

Ankylosing spondylitis (AS) is a highly heritable multifactorial disease; however, the pathophysiological and structural alterations remain largely undefined^1^. The genetics of AS suggest that *HLA-B27* alleles represent the highest risk burden. Although more than 80% of cases are *HLA-B27* allele positive, only a minority (approximately 8%) of *HLA-B27* carriers actually develop AS^2^,^3^,^4^,^5^,^6^. The identification of risk loci through genome-wide association studies (GWAS) in AS have revealed ~30 non-HLA loci. Although, a few genes reported to be associated with extra-articular manifestations of AS (i.e., Crohn’s disease (CD) and ulcerative colitis (UC)), a large number of genes showed an AS-specific association. Collectively, these loci explain only a fraction of the total genetic burden of AS^4^,^5^. Elucidating this missing heritability will help to better define the full spectrum of the risk factors for AS^5^,^6^,^7^,^8^. This demands investigation of alternative approaches besides DNA sequence level variation. Capturing loci that are highly interconnected with AS disease pathogenesis through protein interactions, epistasis or other regulatory mechanisms represent one such approach which holds promise.

The risk loci with small effect sizes for immune mediated diseases poses a great challenge to explain the total genetic burden^7^,^9^. GWAS only investigate a single type of variant and also lacks the power and scope to detect other direct or indirect risk loci that are differentially regulated at the mRNA and protein level. Recently, new approaches are being applied in the field of neuropsychiatry to identify indirect associations through a variant’s effect at the mRNA and protein level through interaction networks and pathway enrichment analyses^10^,^11^,^12^,^13^. A few previous attempts were made with immune mediated diseases to detect disease relevant pathways and to capture risk loci through the interconnectedness of genes assessed by protein-protein interactions^9^,^14^,^15^,^16^,^17^. Importantly, those studies provided information either from a single interactome dataset with a limited number of interactions^15^ or investigated a limited number of autoimmune risk genes^14^,^17^.

In this study, for non-HLA autoimmune disease risk loci, the aim is to demonstrate the apparent similar patterning of the underlying molecular pathways and networks through the use of large scale datasets. We have employed multiple comprehensive integrated genomic approaches (Fig. S1) comprising multiple independent datasets (i.e., population scale RNA-seq, human developmental protein expression profile, large protein co-expression interactome network and pathway enrichment) to better capture the convergence of non-HLA AS risk loci.

This study revealed that non-HLA autoimmune disease risk genes are sensitive to accumulation of deleterious mutations; that such genes have higher protein expression in hematopoietic cells using human developmental mass spectrometry protein expression data; that pathway analysis showed significant shared pathway enrichment of these genes; and that protein-protein interaction (PPI) network analysis showed these non-HLA autoimmune disease risk genes are highly interconnected on functional gene networks. As a proof-of-concept, we have also provided evidence supporting novel regulatory candidate genes that are highly interconnected with AS risk loci and may contribute to disease pathogenesis.

## Results

### Overlap of AS risk genes

Analyses were performed on 407 curated genes from nine immune mediated diseases (AS, crohn’s disease (CD), psoriasis (PS), ulcerative colitis (UC), celiac disease (CeD), multiple sclerosis (MS), primary biliary cirrhosis (PBC), rheumatoid arthritis (RA) and type-1 diabetes (T1D)); (Fig. S1; Table S1). AS risk genes highly overlapped with CD (11 genes) and UC (8 genes); (Fig. S2). A moderate overlap with CeD (5 genes), and psoriasis (PS) (5 genes) was also observed, whereas a minimum overlap was observed between AS and T1D (4 genes), MS (4 genes), and PBC (1 gene). There were eight genes unique to AS, 17 genes that overlapped with at most two immune mediated diseases, and five genes that overlapped with more than two immune diseases (Fig. S2).

### Measure of purifying selection for immune mediated risk genes

**Figure 1:**
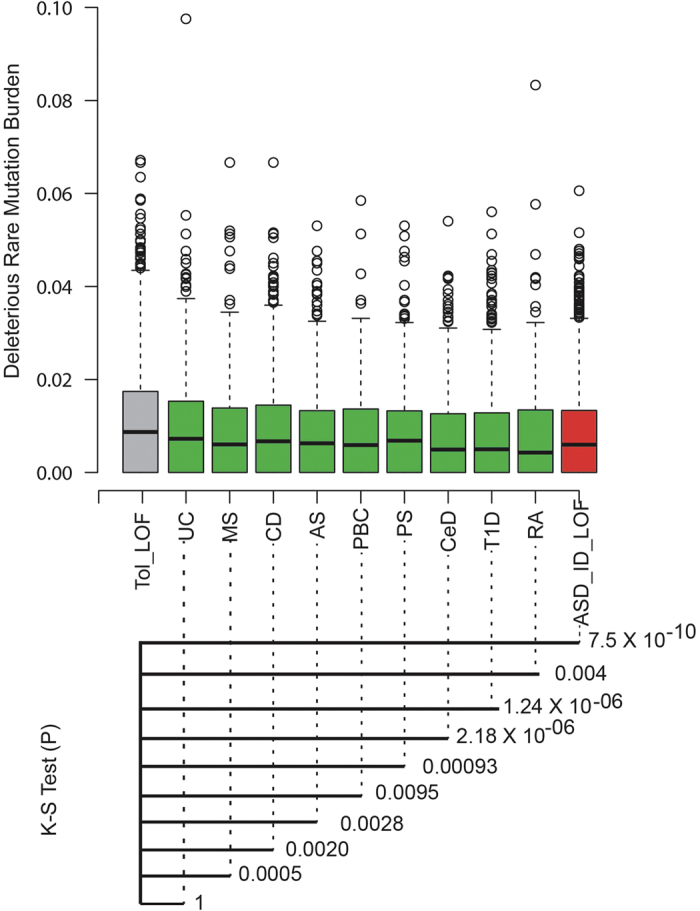
The burden of recent deleterious mutation accumulation. The deleterious mutation burden (rare missense and LOF) for each exon (normalized by exon length) was computed for phenotype (red and green) and non-phenotype tolerated gene sets (grey). The whisker plot shows each bar with minimum and maximum quartiles. The grey bar represents the accumulation of exonic rare deleterious LOF mutations within genes that are tolerated in humans for LOF mutations. The green bar represents the immune mediated disease and the red bar represents genes reported to have deleterious LOF mutations in autism spectrum disorder (ASD). The significance (K-S test p-value) of rare deleterious mutation accumulation for a phenotype was obtained by comparing the burden distribution between tolerated and phenotypic genes.

The concept of purifying selection essentially can be quantified by conducting exon level burden analysis of deleterious mutations for a gene^10^. We have curated a set of genes that have loss of function (LOF) mutations in the general population and do not produce any observable phenotype^18^. Analysis of quantifying purifying selection of each phenotypic gene set comparing tolerated gene sets revealed that immune mediated risk genes were under significant purifying selection pressure, except UC-associated genes. CeD and T1D displayed the most significant deviation, *p* *<* 2.18 × 10^-6^ and *p* *<* 1.24 × 10^-6^, respectively. All other immune mediated disease risk genes also displayed a similar pattern of missense variation accumulation that deviated in comparison with tolerated LOF genes. Phenotypically, these genes were demonstrated to tolerate LOF and can have excessive mutational burden without any untoward phenotypic impact. In contrast, the most significant deviation was observed in autism spectrum disorder (ASD) and intellectual disability (ID) genes that were highly sensitive to deleterious variants (Fig. 1).

### Transcript abundance of immune mediated risk genes

**Figure 2:**
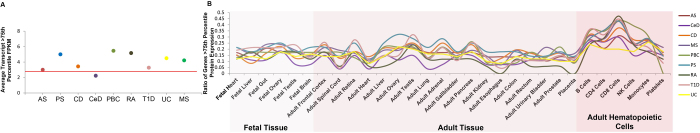
The landscape of protein expression from human developmental tissues. (**A**) The mRNA expression (FPKM) from LCL was computed for gene transcript (>75^th^ percentile expression of all genes in the genome) and the number of transcripts for each gene in the genome was computed. The red line represents the average number of transcripts in the genome that is above threshold. The circle represents transcript abundance above threshold for each disease (colored by disease). (**B**) Each immune mediated risk gene set and the proportion of highly (>75^th^ percentile expression of the genome of all tissue) expressed genes in each tissue (Y-axis). The two developmental periods (fetal and adult) and the tissues are highlighted in different red gradients (X-axis, light to dark red) that correspond to the ratio of genes. Each line represents an immune mediated disease risk gene set and their corresponding ratio of highly expressed genes for a tissue (colored by disease).

To quantify differentially expressed transcripts for each immune mediated disease, the fragment per kilobase mapped (FPKM) was computed for each disease using the genome as background. Transcripts that were highly expressed (i.e., above 75^th^ percentile of the genome) revealed a higher number of transcript abundance for risk genes compared with the genome background. PS, PBC and RA displayed an excess number of highly expressed gene transcripts compared with other disease genes (Fig. 2A). AS, CD and T1D displayed highly expressed transcript abundance close to the genome average with CeD displaying the lowest transcript abundance.

### Developmental human protein expression

Protein expression was analyzed for the immune mediated disease risk genes using 29 histologically normal samples in two developmental periods (i.e., fetal and adult). The overall averaged expression for each immune mediated disease risk gene set revealed evidence of tissue of origin (Fig. S3). Except for T1D, the overall expression was higher in hematopoietic cells compared with other tissues in all disease groups. The averaged expression in tissues indicated that AS genes were highly expressed in adult retina (12.48 average spectra per gene) compared with other organelles (Fig. S3). Similarly, apart from hematopoietic cells, higher protein expression was detected in colon and gallbladder in CeD, pancreas in T1D and PBC, retina in MS and PS, and frontal cortex in CD and UC. Genes associated with RA displayed the weakest protein expression. An analysis was subsequently performed to determine the proportion of the risk genes that are highly expressed (above the 75^th^ percentile of the entire genome) for each tissue (Fig. 2B). The ratio of highly expressed protein revealed that these genes were at least 2-fold more expressed within adult hematopoietic cells, specifically in B-cells, CD4-cells, and CD8-cells compared with other tissues.

### Pathway enrichment analysis

**Figure 3:**
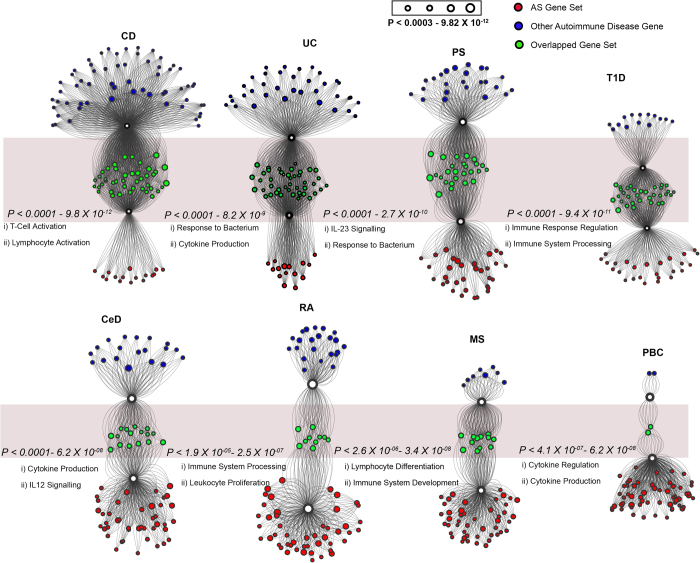
Pathway enrichment of overlapping AS genes. The enrichment analysis of pathways through gene set association was conducted for each gene set. Each node within the network represents a significant gene set belonging to a pathway. The number of edges of a pathway node corresponds to the number of risk genes overlapped with that particular pathway gene set. The size of the node corresponds to the p-value. The mapped green nodes represent overlapping significant pathways between AS (red node) and other immune mediated diseases (blue node). The range of p-values for the overlapping pathways is indicated next to each disease group. The most significant top two pathways that overlap with AS risk genes are displayed below the p-value.

A comprehensive gene set analysis was performed to obtain significantly enriched gene pathways using Fisher’s exact test (FET). After multiple corrections and using a strict cut-off (see method) for each phenotype, significant gene sets, which belong to specific pathways, were obtained (Table S2). The top two significant pathways for AS involved cytokine production (corrected; *p* *<* 5.10 × 10^-5^) and response to bacterium (corrected; *p* *<* 2.24 × 10^-5^). The most significant pathways (after multiple correction test) for all the other immune mediated diseases were: T-cell activation (*p* *<* 1.66 × 10^-8^) for CD, NKT pathway (*p* *<* 6.54 × 10^-6^) for CeD, lymphocyte differentiation (*p* *<* 2.92 × 10^-4^) for MS, IL-23-mediated signaling events (*p* *<* 2.57 × 10^-7^) for PS, regulation of lymphocyte activation (*p* *<* 0.0005) for RA, regulation of immune response (*p* *<* 7.28 × 10^-7^) for T1D, response to molecule of bacterial origin (*p* *<* 2.3 × 10^-6^) for UC, and peptidyl-tyrosine phosphorylation (*p* *<* 0.009) in PBC (Table S2). To identify overlaps, the enriched pathways from all eight immune mediated diseases were compared with pathways significant for AS risk genes. Strikingly, at least 2-fold more overlapping pathways (significant in both phenotypes) were identified between AS and CD, UC, PS, CeD and T1D compared with the other four immune mediated diseases (Fig. 3).

### Protein-protein interaction network

**Figure 4:**
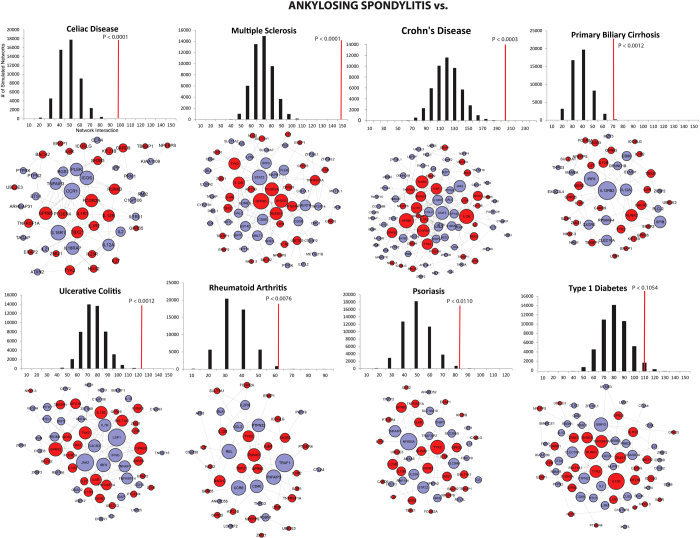
Gene networks associated with immune mediated diseases. Each node represents a risk gene corresponding to AS (red node) or an immune mediated disease (blue node). The edge between the nodes represents interactions. Node size is represented by the number of edges. The top plot displays the permutation test. For each immune mediated gene set, an equal number of genes were randomly selected 50,000 times. The null distribution of the connectivity between the random set and AS gene set is plotted as a histogram (black bras) and the red line represents the original observation. The empirical p-value was computed from the permutation test.

Since shared risk genes will confound the interactions and produce bias association, risk genes associated only with AS were used. This will reveal the interplay of risk loci at the molecular level that have an independent DNA level association. Using this approach, the constructed PPI network revealed significantly (50,000 permutations; empirical *p* *<* 1.6 × 10^-4^, Bonferroni corrected) dense modular connectivity for AS genes with seven other immune mediated disease risk genes (i.e., CD, PS, UC, CeD, MS, RA, and PBC). The highest number of interactions were observed between AS and CD, PS and UC risk genes for non-shared risk genes (Fig. 4).

To capture new candidate genes, we hypothesized that other immune mediated disease risk genes that are highly interconnected with AS risk genes (non-shared) are differentially regulated in AS cases compared with controls. A proof-of-concept study was initiated to test this hypothesis. We randomly picked two AS associated genes, *IL23R* and *NOS2*, and mRNA expression variation was measured between affected and unaffected family members. Expression analysis revealed that these highly interconnected AS genes (i.e., *IL23R* and *NOS2*) had consistently reduced mRNA expression (Fig. S4) in affected individuals compared with unaffected individuals, with the reduction in *NOS2* mRNA expression reaching significance (*p* *<* 0.01).

**Figure 5:**
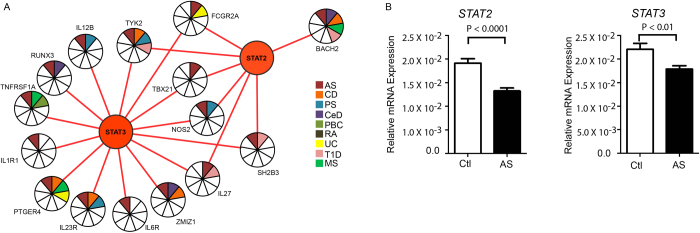
Association of STAT2 and STAT3 with AS. (**A**) Interaction network of *STAT2* and *STAT3* gene with other non-HLA risk genes identified in nine immune mediated diseases. (**B**) Gene expression levels of *STAT3* and *STAT2* in AS cases and controls. A qPCR analysis of patient-derived B-Cell lines showed the relative expression of *STAT2* and *STAT3* genes across the affected (white bar) and unaffected individuals (black bar). Individuals are also grouped according to their clinical diagnosis to compare the expression, normalized to GAPDH only. The bar plot represents the mRNA expression level (Y-axis) and upper and lower limit represented by mean ± SEM. A t-test confirmed significant differences (*p* *<* 0.05) between the groups for both genes.

To better understand the shared pathways and to identify etiological genes, each non-AS gene was ranked according to their contributing connectivity of the inferred network with AS genes. The top two non-AS genes that displayed the greatest connectivity were *STAT3* and *IL12RB2*. *IL12RB2* displayed reduced mRNA expression in AS cases but failed to reach significance (*p* *<* 0.15). In contrast, the mRNA expression of *STAT3* displayed a significant (*p* *<* 0.01) reduction in mRNA expression in AS cases compared with controls. Another gene belonging to the *STAT* family, *STAT2*, was also ranked in the top 10 genes. The mRNA relative expression for *STAT2* was also significantly (*p* *<* 0.0001) reduced in AS cases compared with controls. Interestingly, both *STAT2* and *STAT3* displayed greater connectivity with at least five other immune mediated disease interaction networks (Fig. 5A). Western blot experiments were performed to quantify *STAT2* and *STAT3* protein expression from individuals clinically diagnosed with and without AS (Fig. S5). Similar to mRNA expression, protein expression also revealed consistently low expression in affected individuals compared with unaffected individuals (Fig. S5).

## Discussion

This is the first report demonstrating how an integrated genomics approach can be utilized to infer complex networks, pathways and candidate genes underlying a large set of immune mediated diseases. That AS genes only explain a fraction of the total risk factor^7^,^8^, it is imperative to seek alternative approaches besides DNA sequence level variation to elucidate this missing heritability. Genes associated with AS extra-articular features^3^,^19^,^20^,^21^ may represent shared disease pathways (i.e. co-segregation of CD and UC within AS families^21^). The pleiotropic nature of a few (shared) genes within multiple immune diseases can be captured through GWAS studies (i.e., *GPR65*, *IL23R*, *RUNX3*)^5^, whereas, cellular interconnectedness of non-shared HLA genes are not clear. A statistically significant convergence was demonstrated through overlapping pathway enrichment analysis or protein co-expression network analysis with other immune mediated diseases (T1D and PS), which minimally share risk genes. These findings suggest that AS genes comprise a broader immunity pathway and highlight the significance of investigating non-shared risk genes from other immune diseases to detect an association at the mRNA or protein level.

That the PPI network constructed to reveal the interplay of risk loci at the molecular level displayed significantly dense modular connectivity for AS genes with seven other immune mediated disease risk genes, and that subsequent expression analysis revealed that two of the highly interconnected AS genes (i.e., *IL23R* and *NOS2*) had consistently reduced mRNA expression in affected individuals compared with unaffected individuals, confirmed our hypothesis that other immune mediated disease risk genes that are highly interconnected with AS risk genes (non-shared) are differentially regulated in AS cases compared with controls, and provided the foundation to expand the analysis to identify potential novel candidate genes for AS.

The deleterious mutation burden analysis suggests that all immune risk gene sets under purifying selection compare with tolerated genes in the normal population. That the ratio of highly expressed protein revealed that genes were at least 2-fold more expressed within adult hematopoietic cells, specifically in B-cells, CD4-cells, and CD8-cells compared with other tissues, is consistent with the transcript LCL abundance analysis, and is striking evidence of differential regulation of those genes in adult hematopoietic cells. This finding also supports the adult onset nature for most of the autoimmune diseases. This information can play a vital role for future investigation on models of immune disease mechanisms and drug trials for treatments^22^,^23^,^24^,^25^.

This study provides strong evidence of the pleiotropic effects of individual genes and the importance of Th-17 (T helper 17 cell subset) differentiation in immune disease pathology. That the top ten non-AS genes that displayed the greatest connectivity after each non-AS gene was ranked according to their contributing connectivity of the inferred network with AS genes included *STAT2*, *STAT3* and *IL12RB2*, that the mRNA and protein expression for *STAT2* and *STAT3* was significantly reduced in AS cases compared with controls, and that both *STAT2* and *STAT3* displayed greater connectivity with at least five other immune mediated disease interaction networks, suggests a critical role of the Th-17 signaling pathway in AS pathogenesis. This finding is consistent with genes involved in Th-17 differentiation^25^ and IL-23 signaling reaching a genome-wide level of significance in other related immune diseases^26^,^27^, the prominent role of the Th-17 signaling pathway in the pathogenesis of those related immune diseases^28^,^29^,^30^,^31^, and supports a previous report which nominally associated *STAT3* with AS^3^, and a recent report that *de novo* mutations in *STAT3* have been reported for multi-organ early onset autoimmune disease^32^.

In summary, an extensive and comprehensive gene based analysis was developed and performed to capture the pleotropic nature of immune mediated disease genes. Multiple systematic approaches were employed, which revealed from several lines of evidence, that AS risk genes are indeed biologically clustered with other immune mediated risk genes and that they converge within common immunity pathways. The analysis revealed that: 1) immune mediated disease risk genes (including AS genes) are under significant purifying selection (i.e., less accumulation of deleterious exonic variants) compared with non-phenotypic genes; 2) the risk genes investigated are predominantly expressed (protein) within adult hematopoetic cells; 3) AS risk genes share common pathways with other immune mediated risk genes (i.e., CD, UC, PS and T1D); 4) the interaction networks obtained from AS seed genes were significantly modular with risk genes implicated in global immune conditions than any random set of genes; and 5) the *STAT* gene family and the Th-17 signaling pathway is implicated in AS disease pathogenesis. Our results have several important implications for the interpretation of genes identified through GWAS, specifically for non-shared highly connected perturbed genes.

## Methods

### Recent deleterious mutations within immune mediated risk genes

An extensive analysis was performed using 407 GWAS significant risk genes (Table S1) for the nine immune diseases (AS, CD, PS, UC, CeD, MS, PBC, RA and T1D) to quantitatively assess the burden of deleterious mutations at the exon level as a measure of purifying selection. This was accomplished by analyzing the occurrences of rare missense mutations and loss of function (LOF) mutations for each exon from exome server variants (ESV) comprised of 4,300 European Americans (EA)^33^. A compound exon model was considered to compute rare missense and LOF for each of the immune mediated disease risk genes.

### Burden of recent deleterious mutations within immune mediated risk genes

For whole genome genes, the occurrence of rare missense and LOF mutations annotated in the Genome Analysis Toolkit (GATK) was computed based on RefSeq exon annotation and normalized by exon length. A compound exon model was used where overlapping exons from multiple isoforms were merged into a single breakpoint. For a reference set, 98 genes previously demonstrated to tolerate LOF mutations were curated with each gene harboring at least one LOF in a control population^18^. Given that LOF mutations located within those genes fail to produce an observable phenotype, those genes were treated as a control with the assumption that having rare missense mutations won’t manifest a phenotype. In contrast, risk genes for the nine immune mediated diseases were assumed to be sensitive to rare missense mutations. We also curated 87 genes with LOF mutations identified in autism and intellectual disability from seven published exome/genome sequencing studies^10^,^11^,^34^,^35^,^36^,^37^,^38^. These particular genes were assumed to represent ultra-sensitive rare mutations and consequently should be suppressed from accumulating rare missense mutations. To test the effect of rare missense mutations, a Kolmogorov–Smirnov (K-S) test was performed between the rare missense burden between tolerated LOF genes and the other nine immune mediated diseases.

### Transcript abundance of immune mediated risk genes

We have obtained the pre-computed expression fragment per kilobase mapped (FPKM) for each transcript (from RNA-seq) of the genome from lymphoblastoid cell lines (LCL) derived from 45 individuals comprising the Human Genome Diversity Panel (HGDP)^39^. A total of 216,636 transcripts detected by LCL were annotated exons in Refseq, Ensembl, UCSC, or Gencode databases. The FPKM for a transcript was computed using cufflinks 2.2.0 and normalized^39^. To capture the highly expressed transcript abundance, the average number of transcripts in the genome that were expressed above the 75^th^ percentile in the population was computed. Similarly, the average number of transcripts that were expressed above the 75^th^ percentile was also computed for each immune mediated risk set.

### Human developmental protein expression

The protein expression level for the immune mediated disease risk genes were analyzed using high-resolution genome-wide Fourier-transform mass spectrometry (downloaded from Human Proteome Map) from six human fetal tissues (i.e., heart, liver, gut, ovary, testis, and brain), 18 adult tissues (i.e., frontal cortex, spinal cord, retina, heart, liver, ovary, testis, lung, adrenal, gallbladder, pancreas, kidney, esophagus, colon, rectum, urinary bladder, prostate, and placenta), and six purified primary hematopoietic cells (i.e., B, CD4, CD8, NK, monocytes, and platelets) from histologically normal samples^40^. The data resulted in the identification of proteins encoded by 17,294 genes accounting for approximately 84% of the total annotated protein-coding genes in humans^40^. The average protein expression (i.e., averaged spectral counts per gene per sample) of all genes in the genome for each tissue type was computed. To identify highly expressed tissues (top 25^th^ percentile) for each disease, the ratio of genes for each tissue where the genes were expressed above the 75^th^ percentile cutoff of the entire genome dataset was computed.

### Gene enrichment analysis

The gene sets were derived from the manually-curated gene ontology (GO) (R package, version 2.8.0), pathways from the National Cancer Institute at the National Institutes of Health (NIH; May 30^th^-2013), Kyoto Encyclopedia of Genes and Genomes (KEGG; May 30^th^-2013), and Reactome (June 3^rd^-2013). Large gene sets often represent broad categories with little biological significance (e.g., regulation of a physiological process, zinc ion binding). In contrast, small gene sets are unlikely to produce statistically meaningful results. Consequently, the analysis was limited to terms annotated with 15 to 1,000 genes (mean = 180) from GO, NCI, KEGG and Reactome, yielding 2,729 gene sets from a background of 23,510 genes.

To identify biologically meaningful pathways, we searched for pathways where risk genes were overrepresented for eight immune mediated diseases. To quantify the significance for a given pathway, a Fisher’s exact test (FET) was performed which evaluates the enrichment of genes in a pathway against the genome backgroundconsisting of 23,510 genes. From the null distribution of p-values, an FDR was computed using the Benjamini-Hochberg procedure. A gene-set was considered to be significantly enriched when FET *p* *<* 1.0 × 10^-3^ and FDR *<* 0.01. After obtaining significantly enriched gene sets for each phenotype, an assessment of how many of these gene sets overlap with the significant gene sets obtained from AS was performed.

### Protein-protein interaction network of non-overlapping genes

To investigate the complex PPI interactions between AS and other immune diseases, a total of 407 risk genes were analyzed from nine other complex immune mediated diseases (Table S1). A network-based method was used to infer connectivity between AS genes and the eight other immune mediated disease risk gene sets determined from GWAS. That there were overlapping genes between AS and other immune mediated diseases, indicates that the network was biased by the shared genetic overlaps. Consequently, the immune mediated disease risk genes that overlap with AS were excluded from subsequent analysis. The net result represents a genuine interaction network between non-overlapping genes. Non-shared AS risk genes were used as seeds and inferred interaction networks were compared with other immune mediated diseases risk genes. Using GeneMANIA, which represents the largest resource of gene interaction networks for multiple organisms^41^, the combined interaction networks for humans where the core of the interaction dataset comprised of 6,998,947 pairwise interactions was computed. This set was derived from physical PPI, co-expression or co-localization analyses. Symmetric gene pair interactions were excluded and each gene pair consisted of one degree of connectivity (please see supplementary text for network visualization software details).

### Permutation test

One problem inherent with interaction data is that some genes are well studied and likely to produce more connections compared with other genes^42^,^43^. To eliminate such biases, an exhaustive node connectivity based permutation test was performed for a pair of gene sets. For each pair of gene set (A and B), the genes within set A were comprised of AS risk genes, whereas genes within set B were comprised of risk genes form one of the eight immune mediated diseases. Initial connectivity was inferred based on gene set A and B from the interaction data. An exhaustive permutation test for set B was performed by randomly replacing an equal number of genes in set B (50,000 times, randomly selected from a background of 22,695 protein coding genes). The empirical *p* value was computed by comparing the original connectivity with the connectivity from randomly selected genes using a Bonferroni multiple correction test.

### Q-PCR and Western Blot Analysis

Real-time PCR was performed using TaqMan Gene Expression Assays for *IL12RB2* (Hs00155486_m1), *JAK2* (Hs00234567_m1), *LSP1* (Hs00158886_m1), *NOS2* (Hs01075529_m1), *STAT2* (Hs01013123_m1), *STAT3* (Hs00374280_m1) and *GAPDH* (Hs99999905_m1) - all from Life Technologies (Cat#4331182). Samples were tested as per manufacturer’s instructions using a ViiA 7 (Life Technologies). Triplicate samples were analyzed using the comparative threshold cycle method and samples were expressed normalized to *GAPDH* only (∆CT) or normalized to both *GAPDH* and the Proband (∆∆CT). Results are illustrated as mean ± SEM and statistical analysis was performed using one-way ANOVA or a t-test. Please see supplementary text for cell line culture and RNA extraction.

RIPA lysates were prepared from 4 × 10^6^ BCL per sample. Cells were collected in 15 ml tubes and washed with ×1 with PBS. To each cell pellet, 100 ul of RIPA buffer containing Halt Protease Inhibitor cocktail, Halt Phosphatase Inhibitor cocktail and PMSF[200 uM] (all from Thermo Scientific) was added and tubes rotated on ice for 5 min. Lysates were centrifuged at 15,000 g, for 10 min at 4 ^0^C. Supernatants were transferred to new tubes and stored at −80 ^0^C. Protein determination was performed using a BCA Protein Assay kit (Thermo Scientific). For gel running samples were diluted with water and loading buffer to a final concentration of 1-2 ug Protein/ul and denatured by boiling for 6 min. 20 ug protein samples were loaded per lane on a precast TGX polyacrylamide gels (4-20%, BioRad), along with a lane containing Precision Plus Dual Color Protein Standards (BioRad) and electrophoresed for 1.5 hr at 100 V. Transfer to nitrocellulose was performed for 1hr at 100 V. Blots were blocked with 5%milk/TBS-T for 1 hr, probed with antibodies against *STAT2* (Genscript A01263), *STAT3* (Cel Sign. 9132) or Tubulin (AbCam 44928), followed by overnight incubation at 4 ^0^C on a rocking platform. The blots were then incubated for 1 hr in RT using the appropriate secondary antibody - Peroxidase labelled Goat anti-Rabbit IgG/anti-Mouse IgG. Bands were detected using Immobilon Western Chemiluminescent Subtrate (Millipore) and the ImageQuant LAS 4000 imaging system. The ImageQuant TL program was used to quantitate the density of the bands. Samples were expressed in relative units (RU) of protein, which is the ratio of the specific protein to their corresponding Tubulin. Results are illustrated as mean ± SEM and statistical analysis was performed using a t-test.

## Author Contributions

M.U. and P.R. conceived the project and designed the experiments. M.U. primarily conducted most of the experiments. D.C. and S.M.M.H. helped M.U. conducting wet-lab experiments. M.U., D.O., S.W.S. and P.R. wrote the manuscript.

## Additional Information

**How to cite this article**: Uddin, M. *et al*. Integrated Genomics Identifies Convergence of Ankylosing Spondylitis with Global Immune Mediated Disease Pathways. *Sci. Rep.* **5**, 10314; doi: 10.1038/srep10314 (2015).

## Supplementary Material

Supplementary Information

Supplementary Tables

## References

[b1] Masi A. T. Might axial myofascial properties and biomechanical mechanisms be relevant to ankylosing spondylitis and axial spondyloarthritis? Arthritis Res. Ther. 16, 107, 10.1186/ar4532 (2014). 25166000 PMC4060468

[b2] Choi C. B. *et al.* ARTS1 polymorphisms are associated with ankylosing spondylitis in Koreans. Ann. Rheum. Dis. 69, 582–584, 10.1136/ard.2008.105296 (2010). 19414429

[b3] Danoy P. *et al.* Association of variants at 1q32 and STAT3 with ankylosing spondylitis suggests genetic overlap with Crohn’s disease. PLoS genetics 6, e1001195, 10.1371/journal.pgen.1001195 (2010). 21152001 PMC2996314

[b4] Evans D. M. *et al.* Interaction between ERAP1 and HLA-B27 in ankylosing spondylitis implicates peptide handling in the mechanism for HLA-B27 in disease susceptibility. Nature genetics 43, 761–767, 10.1038/ng.873 (2011). 21743469 PMC3640413

[b5] International Genetics of Ankylosing Spondylitis, C. *et al.* Identification of multiple risk variants for ankylosing spondylitis through high-density genotyping of immune-related loci. Nature genetics 45, 730–738, 10.1038/ng.2667 (2013). 23749187 PMC3757343

[b6] Uddin M. *et al.* UGT2B17 copy number gain in a large ankylosing spondylitis multiplex family. BMC genetics 14, 67, 10.1186/1471-2156-14-67 (2013). 23927372 PMC3751806

[b7] Brown M. A. Progress in the genetics of ankylosing spondylitis. Briefings in functional genomics 10, 249–257, 10.1093/bfgp/elr023 (2011). 21965815

[b8] Visscher P. M., Brown M. A., McCarthy M. I. & Yang J. Five years of GWAS discovery. Am. J. Hum. Genet. 90, 7–24, 10.1016/j.ajhg.2011.11.029 (2012). 22243964 PMC3257326

[b9] Parkes M., Cortes A., van Heel D. A. & Brown M. A. Genetic insights into common pathways and complex relationships among immune-mediated diseases. Nature reviews. Genetics 14, 661–673, 10.1038/nrg3502 (2013). 23917628

[b10] Uddin M. *et al.* Brain-expressed exons under purifying selection are enriched for de novo mutations in autism spectrum disorder. Nature genetics 46, 742–747, 10.1038/ng.2980 (2014). 24859339

[b11] O’Roak B. J. *et al.* Sporadic autism exomes reveal a highly interconnected protein network of de novo mutations. Nature 485, 246–250, 10.1038/nature10989 (2012). 22495309 PMC3350576

[b12] Parikshak N. N. *et al.* Integrative functional genomic analyses implicate specific molecular pathways and circuits in autism. Cell 155, 1008–1021, 10.1016/j.cell.2013.10.031 (2013). 24267887 PMC3934107

[b13] Willsey A. J. *et al.* Coexpression networks implicate human midfetal deep cortical projection neurons in the pathogenesis of autism. Cell 155, 997–1007, 10.1016/j.cell.2013.10.020 (2013). 24267886 PMC3995413

[b14] Stevens A., Meyer S., Hanson D., Clayton P. & Donn R. P. Network analysis identifies protein clusters of functional importance in juvenile idiopathic arthritis. Arthritis Res. Ther. 16, R109, 10.1186/ar4559 (2014). 24886659 PMC4062926

[b15] Hannum G. *et al.* Genome-wide association data reveal a global map of genetic interactions among protein complexes. PLoS genetics 5, e1000782, 10.1371/journal.pgen.1000782 (2009). 20041197 PMC2788232

[b16] Perera G. K. *et al.* Integrative biology approach identifies cytokine targeting strategies for psoriasis. Science translational medicine 6, 223ra222, 10.1126/scitranslmed.3007217 (2014). 24523322

[b17] Rossin E. J. *et al.* Proteins encoded in genomic regions associated with immune-mediated disease physically interact and suggest underlying biology. PLoS genetics 7, e1001273, 10.1371/journal.pgen.1001273 (2011). 21249183 PMC3020935

[b18] MacArthur D. G. *et al.* A systematic survey of loss-of-function variants in human protein-coding genes. Science 335, 823–828, 10.1126/science.1215040 (2012). 22344438 PMC3299548

[b19] Richette P. *et al.* Psoriasis and phenotype of patients with early inflammatory back pain. Ann. Rheum. Dis. 72, 566–571, 10.1136/annrheumdis-2012-201610 (2013). 22679307

[b20] Shivashankar R. *et al.* Incidence of Spondyloarthropathy in patients with ulcerative colitis: a population-based study. J. Rheumatol. 40, 1153–1157, 10.3899/jrheum.121029 (2013). 23678160 PMC3877696

[b21] Thjodleifsson B., Geirsson A. J., Bjornsson S. & Bjarnason I. A common genetic background for inflammatory bowel disease and ankylosing spondylitis: a genealogic study in Iceland. Arthritis and rheumatism 56, 2633–2639, 10.1002/art.22812 (2007). 17665420

[b22] Wang X., Lin Z., Wei Q., Jiang Y. & Gu J. Expression of IL-23 and IL-17 and effect of IL-23 on IL-17 production in ankylosing spondylitis. Rheumatology international 29, 1343–1347, 10.1007/s00296-009-0883-x (2009). 19247658

[b23] Duftner C. *et al.* Preferential type 1 chemokine receptors and cytokine production of CD28- T cells in ankylosing spondylitis. Ann. Rheum. Dis. 65, 647–653, 10.1136/ard.2005.042085 (2006). 16219708 PMC1798130

[b24] Poddubnyy D. A. *et al.* Relation of HLA-B27, tumor necrosis factor-alpha promoter gene polymorphisms, and T cell cytokine production in ankylosing spondylitis – a comprehensive genotype-phenotype analysis from an observational cohort. J. Rheumatol. 38, 2436–2441, 10.3899/jrheum.110130 (2011). 21885496

[b25] Bal A. *et al.* Comparison of serum IL-1 beta, sIL-2R, IL-6, and TNF-alpha levels with disease activity parameters in ankylosing spondylitis. Clinical rheumatology 26, 211–215, 10.1007/s10067-006-0283-5 (2007). 16583185

[b26] Nair R. P. *et al.* Genome-wide scan reveals association of psoriasis with IL-23 and NF-kappaB pathways. Nature genetics 41, 199–204, 10.1038/ng.311 (2009). 19169254 PMC2745122

[b27] Wang K. *et al.* Diverse genome-wide association studies associate the IL12/IL23 pathway with Crohn Disease. Am. J. Hum. Genet. 84, 399–405, 10.1016/j.ajhg.2009.01.026 (2009). 19249008 PMC2668006

[b28] Axtell R. C. *et al.* T helper type 1 and 17 cells determine efficacy of interferon-beta in multiple sclerosis and experimental encephalomyelitis. Nature medicine 16, 406–412, 10.1038/nm.2110 (2010). PMC3042885 20348925

[b29] Wei L., Laurence A., Elias K. M. & O’Shea J. J. IL-21 is produced by Th17 cells and drives IL-17 production in a STAT3-dependent manner. J. Biol. Chem. 282, 34605–34610, 10.1074/jbc.M705100200 (2007). 17884812 PMC2323680

[b30] Sutton C. E., Mielke L. A. & Mills K. H. IL-17-producing gammadelta T cells and innate lymphoid cells. European journal of immunology 42, 2221–2231, 10.1002/eji.201242569 (2012). 22949320

[b31] Cua D. J. & Tato C. M. Innate IL-17-producing cells: the sentinels of the immune system. Nature reviews. Immunology 10, 479–489, 10.1038/nri2800 (2010). 20559326

[b32] Flanagan S. E. *et al.* Activating germline mutations in STAT3 cause early-onset multi-organ autoimmune disease. Nature genetics 46, 812–814, 10.1038/ng.3040 (2014). 25038750 PMC4129488

[b33] Fu W. *et al.* Analysis of 6,515 exomes reveals the recent origin of most human protein-coding variants. Nature 493, 216–220, 10.1038/nature11690 (2013). 23201682 PMC3676746

[b34] Neale B. M. *et al.* Patterns and rates of exonic de novo mutations in autism spectrum disorders. Nature 485, 242–245, 10.1038/nature11011 (2012). 22495311 PMC3613847

[b35] Sanders S. J. *et al.* De novo mutations revealed by whole-exome sequencing are strongly associated with autism. Nature 485, 237–241, 10.1038/nature10945 (2012). 22495306 PMC3667984

[b36] Iossifov I. *et al.* De novo gene disruptions in children on the autistic spectrum. Neuron 74, 285–299, 10.1016/j.neuron.2012.04.009 (2012). 22542183 PMC3619976

[b37] Jiang Y. H. *et al.* Detection of clinically relevant genetic variants in autism spectrum disorder by whole-genome sequencing. Am. J. Hum. Genet. 93, 249–263, 10.1016/j.ajhg.2013.06.012 (2013). 23849776 PMC3738824

[b38] O’Roak B. J. *et al.* Exome sequencing in sporadic autism spectrum disorders identifies severe de novo mutations. Nature genetics 43, 585–589, 10.1038/ng.835 (2011). 21572417 PMC3115696

[b39] Martin A. R. *et al.* Transcriptome sequencing from diverse human populations reveals differentiated regulatory architecture. PLoS genetics 10, e1004549, 10.1371/journal.pgen.1004549 (2014). 25121757 PMC4133153

[b40] Kim M. S. *et al.* A draft map of the human proteome. Nature 509, 575–581, 10.1038/nature13302 (2014). 24870542 PMC4403737

[b41] Zuberi K. *et al.* GeneMANIA prediction server 2013 update. Nucleic acids research 41, W115–122, 10.1093/nar/gkt533 (2013). 23794635 PMC3692113

[b42] Halldorsson B. V. & Sharan R. Network-based interpretation of genomic variation data. J. Mol. Biol. 425, 3964–3969, 10.1016/j.jmb.2013.07.026 (2013). 23886866

[b43] Koh G. C., Porras P., Aranda B., Hermjakob H. & Orchard S. E. Analyzing protein-protein interaction networks. J. Proteome. Res. 11, 2014–2031, 10.1021/pr201211w (2012). 22385417

