# Supplementary material for: Integrated Genomics Identifies Convergence of Ankylosing Spondylitis with Global Immune Mediated Disease Pathways
Source: Sci Rep. 2015 May 18;5:10314. doi: 10.1038/srep10314 (PMC4434845; doi:10.1038/srep10314)
Supplement: Supplementary Information [file srep10314-s1.doc]

**Supplementary Information: Integrated Genomics Identifies Convergence of Ankylosing Spondylitis with Global Immune Mediated Disease Pathways**

Mohammed Uddin^1^, Dianne Codner^2^, S M Mahmud Hasan^2^, Stephen W Scherer^1,3,4^, Darren D O’Rielly^2^, Proton Rahman^2^

^1^ Genetics and Genome Biology, The Hospital for Sick Children, Toronto, Ontario, Canada

^2^ Faculty of Medicine, Memorial University, Newfoundland, St. John’s, Canada

^3^ McLaughlin Centre, University of Toronto, Toronto, Ontario, Canada

^4^ Department of Molecular Genetics, University of Toronto, Ontario, Canada

**Funding Sources:** Atlantic Canada Opportunity Agency – Atlantic Innovation Fund (ACOA-AIF), Canadian Institute of Health Research (CIHR), Research and Development Corporation (RDC), Newfoundland and Labrador, and Spondyloarthritis Consortium of Canada (SPARCC).

**Please send all correspondence to:**

Proton Rahman, MD FRCPC

Professor of Medicine and Rheumatology

Memorial University

154 LeMarchant Rd

St. John's, Newfoundland

Canada A1C 5B8

Phone: 709 777 5736

Fax: 709 777 5212

Cell: 709 682 8936

Email: [prahman@mun.ca](mailto:prahman@mun.ca)

**Supplementary Text:**

**Visualization of Enrichment Map**

For visualizing the protein interaction network, Cytoscape network software v.2.8.3 was used. Each circular node represented a gene associated with a phenotype and the connection between a pair of nodes represented an interaction. The node size was proportional to the number of connections, and the constructed network was colored red for AS genes and blue for any other immune mediated genes.

Visualization of the gene set and pathway enrichment analysis was also performed using the Cytoscape network software v.2.8.3. For gene sets associated with pathways that were significantly enriched for a phenotype (described above), a network was constructed where each gene-set associated with a pathway was a node, with the edges representing gene overlap between sets (pathway and immune mediated risk genes). The node size was proportional to the enrichment significant p-value (Benjamini-Hochberg) for each phenotype. The nodes are pathways that were colored red for AS enrichment analysis and blue for other immune mediated disease enrichment analysis. The overlapping gene-sets between AS and an immune mediated disease were represented in green color. For the overlapping node size, the largest p-value from a pair of p-values between the two significant values was used.

**Sample Collection**

A large multiplex AS family from Newfoundland was identified for this study. All members of the multiplex family were “native Newfoundlanders” of North European ancestry where ten members participated in the study. Each individual was assessed clinically including imaging studies. As defined by the 1984 modified New York criteria, six members were diagnosed with AS while the remaining family members were not affected. We have obtained informed consent for participation in the study from participants or, where participants are children, a parent or guardian. All human subjects reported in this manuscript have been approved by the Memorial University of Newfoundland Human Investigation Committee.

**B Cell Lines (BCL)**

Acid Citrate dextrose anti-coagulated whole blood was collected from the members of the family (approved by Memorial University research and ethics board). Whole blood was diluted with PBS, layered over Ficoll-Paque Plus (GE Healthcare) and centrifuged at 400g for 30min. The interface containing mononuclear cells was transferred to a new tube and washed with RPMI (containing antibiotics). Cell pellets were resuspended with 2.5mls RPMI 1640, complete medium (CM) supplemented with 10% heat inactivated FBS, Penicillin/Streptomycin (100U/ml, 100ug/ml), HEPES (10mM), Sodium Pyruvate (1mM), L-glutamine (2mM) and Fungizone (1.4ug/ml); all supplied by Invitrogen, along with cyclosporine (0.1ug/ml), and B958 supernatant (2.5mls) and placed in a 25cm^2^ flask and incubated in a 5% CO_2_, 37^0^C, humidified incubator. Cells were observed microscopically for growth and fed twice weekly or as required.

**RNA**

Approximately 4x10^6^ BCL were centrifuged and resuspended in 0.5ml Trizol and RNA was extracted as per Manufacturer’s protocol. Briefly, Trizol was added to cell pellets, pipetted to lyse the cells and incubated for 5min at RT. Chloroform (100ul) was added, mixed vigorously and incubated for 3min at RT. Samples were centrifuged at 15,000g for 10min at 4^0^C. The upper aqueous phase was transferred to a clean tube and RNA precipitated with an equal volume of 100% isopropanol. Tubes were inverted to mix, incubated for 10min at RT then centrifuged at 15,000g for 10min at 4^0^C. The supernatant was discarded, pellets washed with 1ml 75% ethanol and centrifuged at 15,000g for 5min at 4^0^C. The supernatant was removed and pellets air dried for 5min at RT. RNA was dissolved in Molecular Biology grade water. RNA samples were treated with Turbo DNA-free (Ambion) to remove possible DNA/DNAse contamination. Quantification of samples was performed on a Nanodrop 2000 (Thermo Scientific). cDNA was prepared using the High Capacity cDNA Reverse Transcriptase Kit as per manufacturer’s instructions (Applied Biosystems).

**Figure and Tables**

**
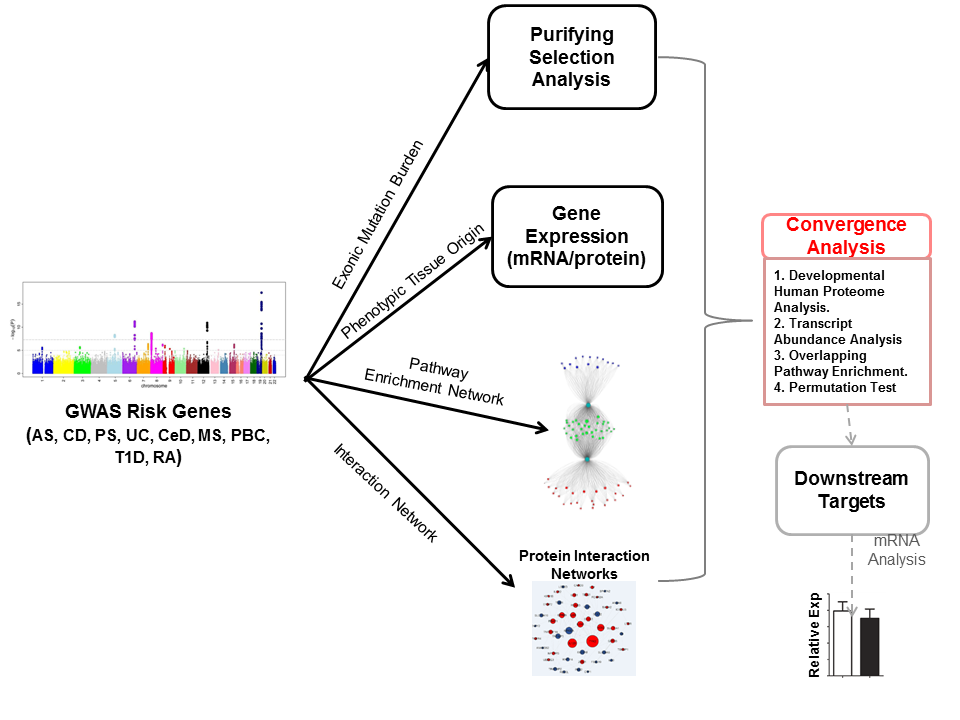
**

**Figure S1.**  Schematic diagram of integrated genomics analyses. The sequential steps of the genome-wide association risk gene analyses involved purifying selection quantification, expression (both mRNA and protein), pathway enrichment, and protein interaction network. Each component used AS risk genes as a reference or seed to quantify the pattern of association. The information from protein network modularity was used to infer candidate genes. As a proof of principle, mRNA and protein level expression was quantified in a small cohort of cases and controls.

**
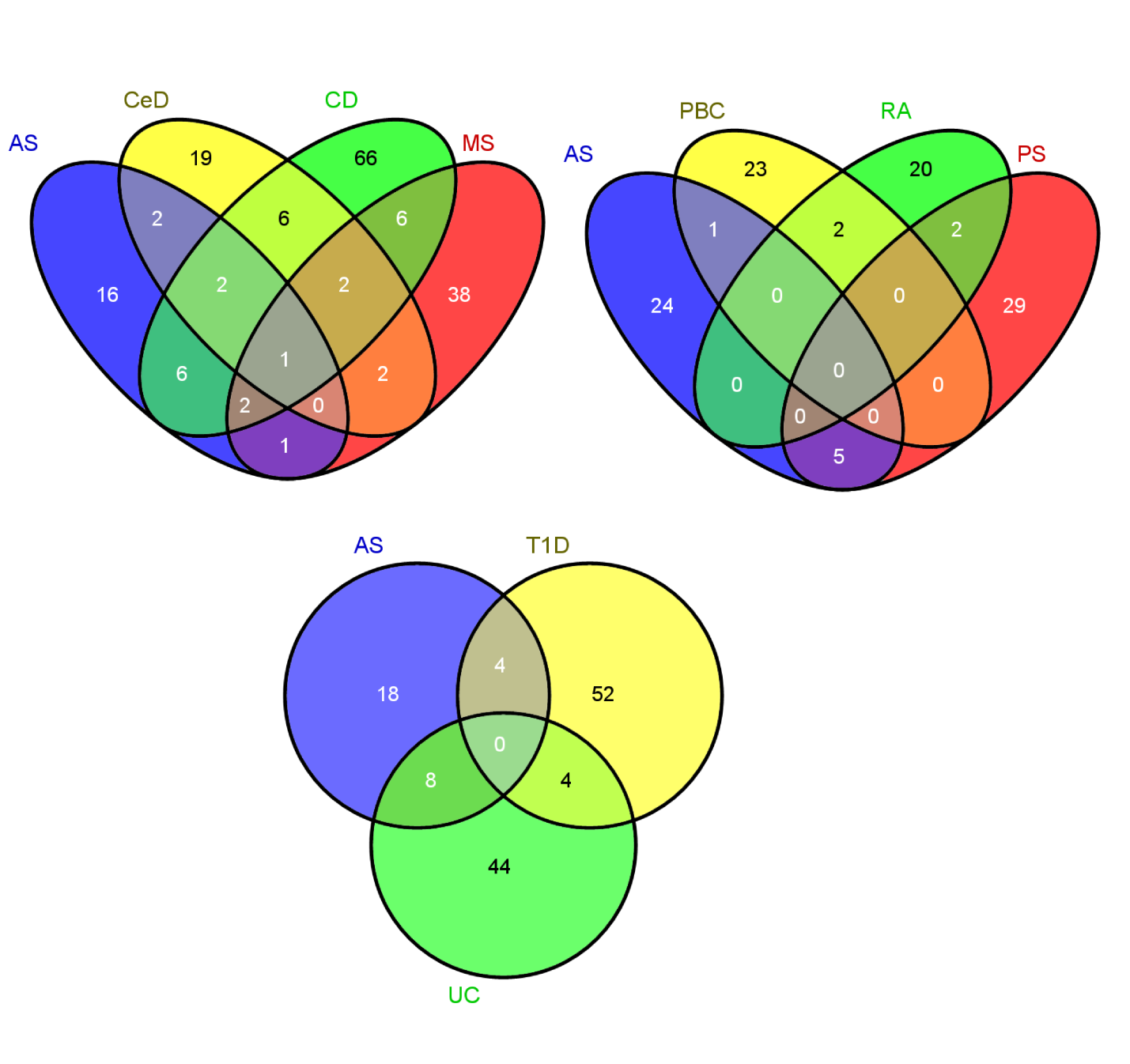
**

**Figure S2.** Risk genes overlapping with AS. The Venn diagrams illustrate overlapping risk genes (identified in genome-wide association studies) between AS or eight other immune mediated diseases. The genetic overlap between AS and other immune mediated diseases reveal that AS-specific genes as well as genes shared between the immune mediated diseases.

**
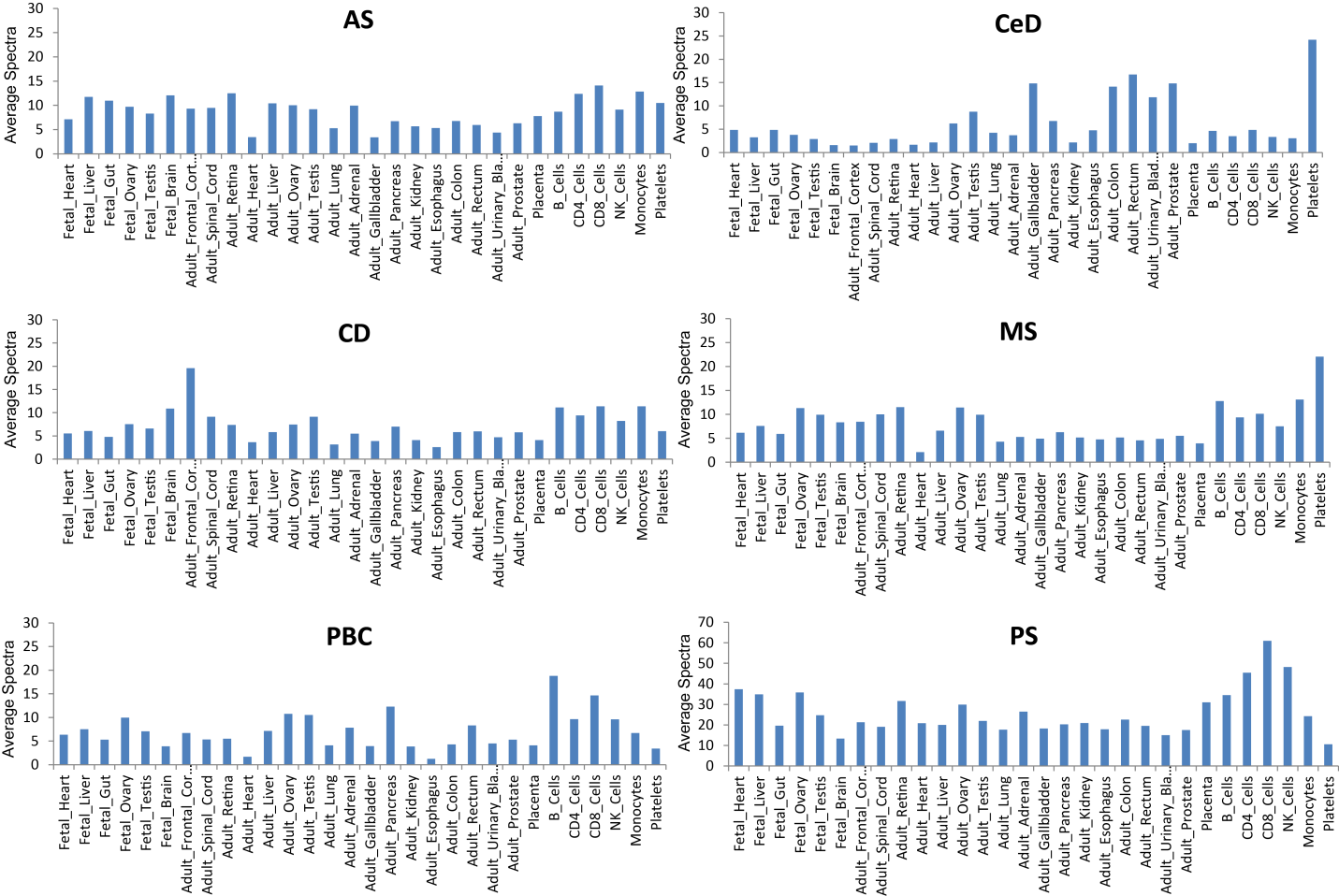

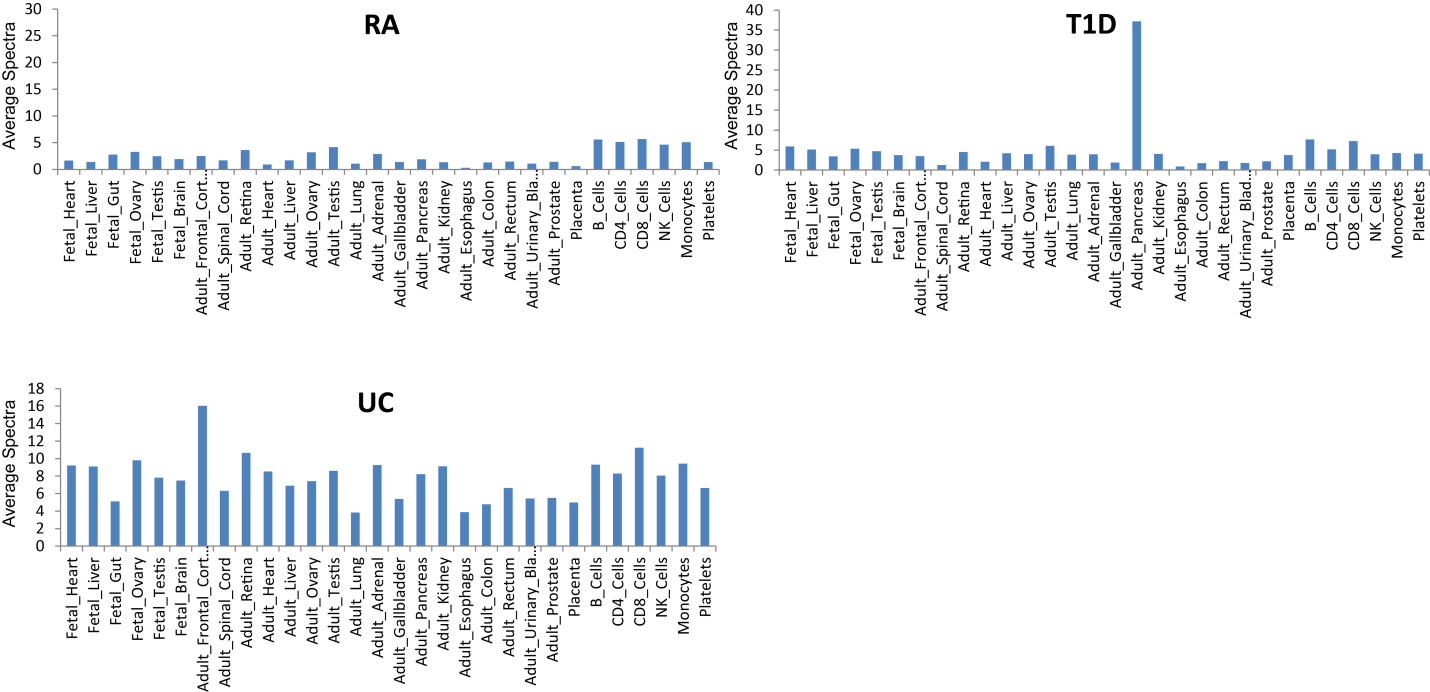
**

**Figure S3.** Average protein expression in developmental human tissues. The average protein expression (averaged spectral counts per gene per sample) for each immune mediated disease risk gene set is plotted for each tissue from six human fetal tissues (heart, liver, gut, ovary, testis, and brain) and 18 adult tissues (frontal cortex, spinal cord, retina, heart, liver, ovary, testis, lung, adrenal, gallbladder, pancreas, kidney, esophagus, colon, rectum, urinary bladder, prostate, and placenta) and six purified primary hematopoietic cells (B, CD4, CD8, NK, monocytes, and platelets) from histologically normal samples.


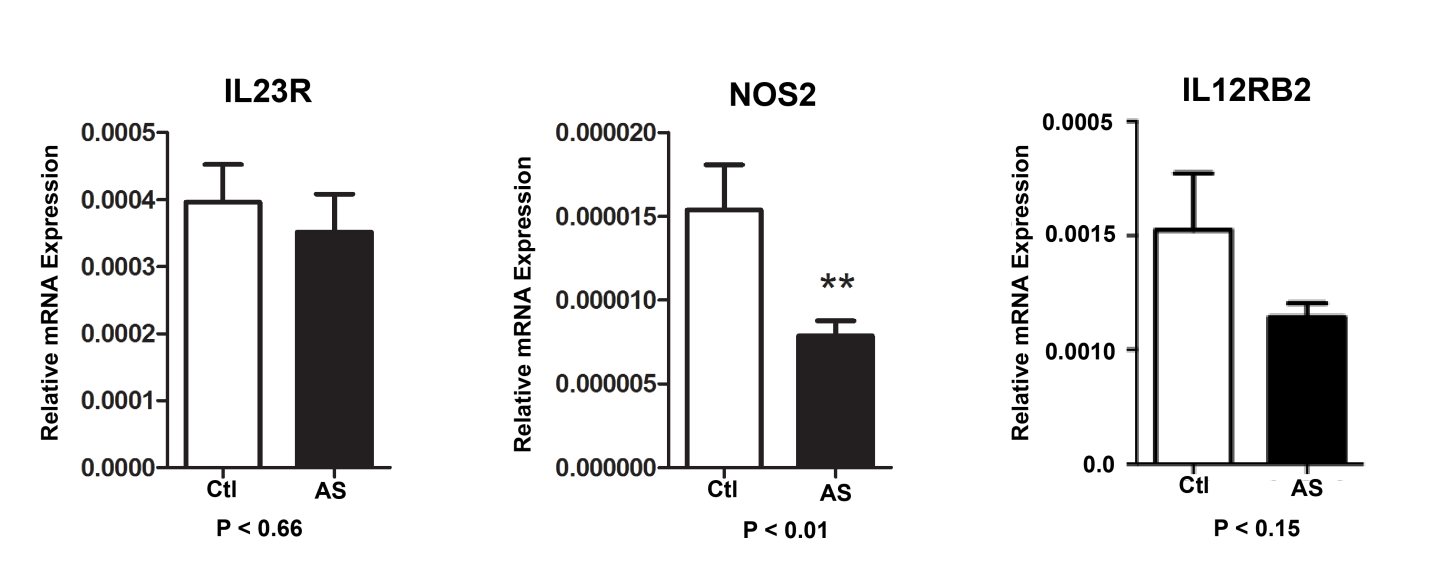


**Figure S4.** *IL23R, NOS2* and *IL12RB2* gene expression in individuals clinically diagnosed with and without AS. QPCR analysis of patient-derived B-Cell lines showed the relative expression of *IL23R*, *NOS2* and *IL12RB2* across the affected and unaffected individuals. Individuals were also grouped according to their clinical diagnosis to compare the expression, normalized to GAPDH only. The bar plot represents the expression level and upper and lower limit represented by mean±SEM. A t-test with *p*<0.05 was considered to be significant between the groups for all 3 genes.

**
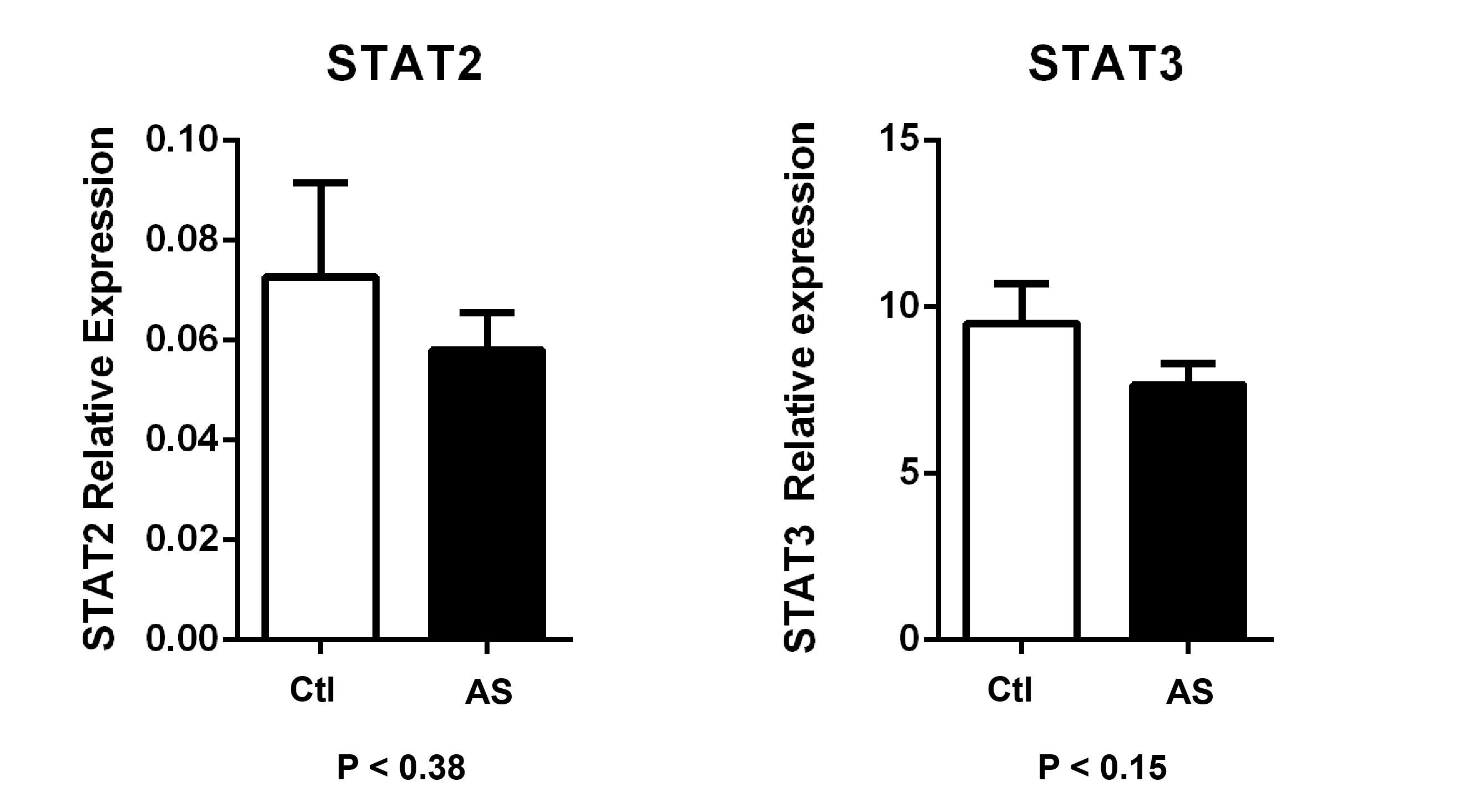
**

**Figure S5.** Western blot protein expression. Similar to mRNA expression analysis, *STAT2* and *STAT3* protein expression showed reduced expression (not significant) in affected compare with unaffected individuals. Blots are representative of triplicate experiments in which Ripa lysates from patient-derived B-cell lines of individuals were tested by western blot analysis. Densitometric analysis was performed to determine the level of *STAT2* and *STAT3* expression relative to tubulin as a loading control. Results are expressed as mean±SEM and analyzed by t-test.

**Table S1.** Gene set from SNP genome-wide association studies that crossed the genome-wide significance threshold (*P <* 1.0 X 10-^07^) curated for 9 immune mediated diseases that are.

| **Disease** | **Genes (cross GWAS threshold)** |
| --- | --- |
| Ankylosing Spondylitis (AS) | 30 |
| Rheumatoid Arthritis (RA) | 24 |
| Psoriasis (PS) | 36 |
| Crohn’s Disease (CD) | 91 |
| Celiac Disease (CeD) | 34 |
| Multiple Sclerosis (MS) | 50 |
| Primary Biliary Cirrhosis (PBC) | 26 |
| Ulcerative Colitis (UC) | 56 |
| Type 1 Diabetes (T1D) | 60 |

**References**

1. MacArthur DG, Balasubramanian S, Frankish A, Huang N, Morris J, et al. (2012) A systematic survey of loss-of-function variants in human protein-coding genes. *Science* 335: 823-828.

2. Neale BM, Kou Y, Liu L, Ma'ayan A, Samocha KE, et al. (2012) Patterns and rates of exonic de novo mutations in autism spectrum disorders. *Nature* 485: 242-245.

3. Sanders SJ, Murtha MT, Gupta AR, Murdoch JD, Raubeson MJ, et al. (2012) De novo mutations revealed by whole-exome sequencing are strongly associated with autism. *Nature* 485: 237-241.

4. Iossifov I, Ronemus M, Levy D, Wang Z, Hakker I, et al. (2012) De novo gene disruptions in children on the autistic spectrum. *Neuron* 74: 285-299.

5. Jiang YH, Yuen RK, Jin X, Wang M, Chen N, et al. (2013) Detection of clinically relevant genetic variants in autism spectrum disorder by whole-genome sequencing. Am J Hum Genet 93: 249-263.

6. Uddin M, Tammimies K, Pellecchia G, Alipanahi B, Hu P, et al. (2014) Brain-expressed exons under purifying selection are enriched for de novo mutations in autism spectrum disorder. *Nat Genet* 46: 742-747.

7. O'Roak BJ, Deriziotis P, Lee C, Vives L, Schwartz JJ, et al. (2011) Exome sequencing in sporadic autism spectrum disorders identifies severe de novo mutations. *Nat Genet* 43: 585-589.

8. O'Roak BJ, Vives L, Girirajan S, Karakoc E, Krumm N, et al. (2012) Sporadic autism exomes reveal a highly interconnected protein network of de novo mutations. *Nature* 485: 246-250.

9. Martin AR, Costa HA, Lappalainen T, Henn BM, Kidd JM, et al. (2014) Transcriptome sequencing from diverse human populations reveals differentiated regulatory architecture. *PLoS Genet* 10: e1004549.

10. Halldorsson BV, Sharan R (2013) Network-based interpretation of genomic variation data. *J Mol Biol* 425: 3964-3969.

11. Koh GC, Porras P, Aranda B, Hermjakob H, Orchard SE (2012) Analyzing protein-protein interaction networks. *J Proteome* Res 11: 2014-2031.
